# Improvement of thermoalkaliphilic laccase (CtLac) by a directed evolution and application to lignin degradation

**DOI:** 10.1007/s00253-022-12311-4

**Published:** 2022-12-07

**Authors:** Youri Yang, Sunil Ghatge, Hor-Gil Hur

**Affiliations:** 1 School of Earth Sciences and Environmental Engineering, Gwangju Institute of Science and Technology (GIST), Gwangju, 61005 Republic of Korea; 2 GREEN BIO Co. Ltd, Gwangju, 61005 Republic of Korea

**Keywords:** Thermoalkaliphilic laccase, Random mutagenesis, Oxygen consumption, Vanillin, Lignin degradation

## Abstract

**Abstract:**

Thermoalkaliphilic laccase (CtLac) from the *Caldalkalibacillus thermarum* strain TA2.A1 has advantageous properties with potential industrial applications, such as high enzyme activity and stability at 70 °C and pH 8.0. In the present study, a directed evolution approach using a combination of random and site-directed mutagenesis was adopted to enhance the laccase activity of CtLac. Spectrophotometric assay and real-time oxygen measurement techniques were employed to compare and evaluate the enzyme activity among mutants. V243 was targeted for site-directed mutagenesis based on library screening. V243D showed a 25–35% higher laccase activity than wild-type CtLac in the spectrophotometric assay and oxygen consumption measurement results. V243D also showed higher catalytic efficiency than wild-type CtLac with decreased *K*_m_ and increased *k*_cat_ values. In addition, V243D enhanced oxidative degradation of the lignin model compound, guaiacylglycerol-β-guaiacyl ether (GGGE), by 10% and produced a 5–30% increase in high-value aldehydes than wild-type CtLac under optimal enzymatic conditions (i.e., 70 °C and pH 8.0). Considering the lack of protein structural information, we used the directed evolution approach to predict Val at the 243 position of CtLac as one of the critical amino acids contributing to the catalytic efficiency of the enzyme. Moreover, it found that the real-time oxygen measurement technique could overcome the limitations of the spectrophotometric assay, and apply to evaluate oxidase activity in mutagenesis research.

**Key points:**

• *CtLac was engineered for enhanced laccase activity through directed evolution approach*

• *V243D showed higher catalytic efficiency (k*_*cat*_*/K*_*m*_*) than wild-type CtLac*

• *V243D produced higher amounts of high-value aldehydes from rice straw than wild-type CtLac*

**Supplementary Information:**

The online version contains supplementary material available at 10.1007/s00253-022-12311-4.

## Introduction

As an oxidoreductase belonging to multicopper-containing oxidases, laccase has been considered a “green” catalyst because of its characteristics in catalyzing the single-electron oxidation of substrates to produce the corresponding reactive radicals, which can undergo oxidative coupling and further polymerization or depolymerization reactions (Riva 2006). The catalytic reaction of laccase is assisted by a four-copper network cluster, that is, type 1 (T1), type 2 (T2), and type 3 (T3), where catalytic reactions take place, such as substrate oxidation, electron transfer, and oxygen reduction (Riva 2006; Yang et al. 2017). During the overall reactions of laccase, oxygen takes electrons as the final electron acceptor and is reduced to water while the active-site copper is oxidized. Therefore, water is produced only as a byproduct of the enzyme reaction. Laccase has a broad substrate spectrum ranging from simple phenolic and nonphenolic compounds to complex aromatic polymers, such as lignin (Agustin et al. 2021; Arregui et al. 2019). These attractive characteristics mean that laccase differs from other oxidase enzymes in industrial applications because the usual co-factors, such as NADH and metal ions, are not needed.

Laccase has been used in diverse industrial fields, such as food, textiles, organic synthesis, and bioremediation (Arregui et al. 2019; Senthivelan et al. 2016; Yang et al. 2017). In addition, laccase has been considered a significantly ecofriendly alternative to harmful chemicals, such as sodium hypochlorite, in the denim-bleaching process in the textile industry. According to various commercial demands, global industries in Denmark, the USA, India, and Hong Kong have produced bleaching products by formulating laccase, redox mediator, and buffer system for bleaching dyes (Rodríguez-Couto 2012). These products are generally used to bleach indigo dyes in denim textiles. In addition, laccase could be applied in lignin polymerization and depolymerization to obtain lignin-based phenolic compounds (Agustin et al. 2021; Arefmanesh et al. 2022; Munk et al. 2015).

Lignin is a heterogeneous, branched, and amorphous aromatic polymer composed of three representative phenylpropanoid subunits (*p*-coumaryl, coniferyl, and sinapyl alcohol) with various C-O and C–C linkages, such as β-aryl ether, phenylcoumaran, and pinoresinol (Behling et al. 2016; Roth and Spiess 2015). Furthermore, lignin is the second most abundant natural biopolymer on Earth after cellulose; therefore, it could be used as a renewable substitute for fossil-based resources (Agustin et al. 2021). However, many attempts have been made to depolymerize lignin using physical, chemical, catalytic, biological, and enzymatic strategies (Abdelaziz et al. 2016; Katahira et al. 2016; Patwardhan et al. 2011; Xu et al. 2018). Among these various methods, ligninolytic enzymes, such as laccase, could be used to depolymerize lignin under much milder reaction conditions in ecofriendly manners compared with other treatments.

Rational and directed evolution mutagenesis are two strategies to improve enzyme properties. The rational mutagenesis mostly requires the accessibility of the protein structure and the relationships between the enzyme’s structure and function to tailor the desired enzyme properties, which is a very informative-intensive approach (Bornscheuer and Pohl 2001). Meanwhile, the directed evolution approach does not require information about protein structure but requires considerable effort in screening thousands of colonies from mutant libraries (Pardo and Camarero 2015). Therefore, the proper screening method is crucial in differentiating and selecting the best mutant from numerous mutants in the directed evolution approach. Spectrophotometric assay has been commonly used to determine the activity of oxidase enzymes by indirectly measuring the absorbance of produced chromogens at a specific wavelength. Even though this assay is simple and easy to perform, it cannot measure enzyme activity in real-time. In other words, only a specific form of product can be detected at a given wavelength, even though various oxidized products continuously could be made until the enzyme is active. Therefore, real-time oxygen measurement should be considered to measure directly oxidase activity, especially to compare activities among mutants in mutagenesis study.

Several attempts have been made to engineer laccase enzyme to enhance their capabilities, such as catalytic efficiency and enzyme stability, via rational and/or directed evolution mutagenesis (Chen et al. 2017; Madzak et al. 2006; Pardo and Camarero 2015; Zhang et al. 2018). These attempts have primarily been focused on the engineering of fungal-origin laccase because of its limited activities, such as low pH dependence (2.0—4.0) (Torres-Salas et al. 2013;

Yin et al. 2019). For example, the catalytic property of fungal laccase from *Trametes versicolor* was improved by site-directed mutagenesis, which showed a threefold increase in *k*_cat_ than the wild-type and the shift of optimal pH from 3.4 to 4.8 (Madzak et al. 2006). In addition, the high-redox-potential laccases from basidiomycete white rot fungi were engineered to increase the redox potential of the T1 copper-binding site, in addition to thermal and acidic pH stability by combining computer-guided rational and directed evolution mutagenesis (Mateljak et al. 2019).

The industrial application of enzymes requires robust characteristics with high enzyme activity and stability under harsh operational conditions, such as high temperature and pH (Pardo and Camarero 2015). Therefore, bacterial laccase with a high resistance toward broad ranges of temperatures and pH would be more advantageous in industrial applications. A novel laccase from thermoalkaliphilic bacterium *Caldalkalibacillus thermarum* strain TA2.A1, CtLac, was well characterized in our previous study. CtLac has a high laccase activity at 70 °C and pH 8.0, interestingly, it is highly thermostable at 60–80 °C for 24 h of reaction (Ghatge et al. 2018). Moreover, it depolymerized lignin by releasing high-value benzaldehyde chemicals, such as vanillin (Yang et al. 2019). Based on these versatile properties, CtLac is worth to be engineered to enhance enzyme activity for industrial application.

In the present study, we induced the directed evolution of CtLac using a combination of random and site-directed mutagenesis to enhance laccase activity. We employed two methods for screening mutants, namely, spectrophotometric assay and real-time oxygen measurement, and compared the enhanced laccase activity with wild-type CtLac. We obtained a mutant with a higher laccase activity and applied it to degrade the lignin model compound guaiacylglycerol-β-guaiacyl ether (GGGE) and rice straw as a representative lignocellulosic biomass abundant in agriculture. The present study found a critical amino acid responsible for enhanced enzyme activity of CtLac in the absence of protein structure information with further potential use in the increased production of high-value phenolic compounds from lignin.

## Materials and methods

### *Escherichia coli* strains, plasmid, and chemicals

*E. coli* strain XL10-Gold (Tet^r^Δ (*mcrA*)*183 Δ*(*mcrCB*-*hsdSMR****-****mrr*)*173 endA1 supE44 thi*-*1 recA1 gyrA96 relA1 lac* Hte [F′ proAB *lacI*^q^*ZΔM15* Tn*10* (Tet^r^) Amy Cam^r^]) and *E. coli* strain BL21(DE3) (F^−^ *dcm ompT hsdS*(r_B_^−^ m_B_^−^) *gal* λ(DE3)) were purchased from Agilent Technology (Santa Clara, CA). *E. coli* strain DH5α (*fhuA2 Δ*(*argF-lacZ*)*U169 phoA glnV44 Φ80 Δ*(*lacZ****)****M15 gyrA96 recA1 relA1 endA1 thi-1 hsdR17*) was purchased from New England BioLabs (Ipswich, MA). The protein expression vector pET28a( +) was purchased from Novagen (Kenilworth, NJ).

Recombinant plasmid pECtLac was constructed in our laboratory (Ghatge et al. 2018) and used as a template for random mutagenesis. 2,6-dimethoxyphenol (2,6-DMP) and GGGE were purchased from Sigma-Aldrich (St. Louis, MO) and TCI Chemicals (Tokyo, Japan). All organic solvents (i.e., acetonitrile, ethanol, ethyl acetate, methanol) were of high-purity high-performance liquid chromatography (HPLC) grade and purchased from Thermo Fisher Scientific (Waltham, MA).

### Library construction by random mutagenesis

We constructed recombinant plasmid pECtLac in our previous study, which was used as a template for random mutagenesis in the current study (Ghatge et al. 2018). Error-prone polymerase chain reaction (PCR) was performed to introduce random mutations into CtLac within the pECtLac plasmid at low mutation frequency using the GeneMorph II EZClone domain mutagenesis kit (Agilent Technology) according to the manufacturer’s protocol. The following primers were used for PCR: Lac-F 5′ACGTACATATGAAACGGATTTTAACACTAGTTCTTC3′ and Lac-R 5′GATACCTCGAG**TTA**TTCAGGTTTGTTCGGGATG3′. The restriction sites of *Nde*I and *Xho*I are underlined and the stop codon is indicated in bold letters. The PCR conditions were followed by segment 1 (95 °C for 2 min), 30 cycles of segment 2 (95 °C for 30 s, 53 °C for 30 s, and 72 °C for 1 min 30 s), and segment 3 (72 °C for 10 min). The resulting PCR products were used as megaprimers in EZClone reaction for the ligation with pET28a( +). *E. coli* strain XL10-Gold was transformed by the resulting plasmids. The plasmid library was prepared from the obtained transformants and retransformed into expression host *E. coli* strain BL21(DE3) followed by protein induction and laccase activity screening using 2,6-DMP as a substrate.

### Library screening

Individual clones of *E. coli* strain BL21(DE3) carrying mutated plasmid were randomly picked and grown overnight in 3 mL of Luria–Bertani (LB) broth supplemented with 50 μg/mL kanamycin at 37 °C and 200 rpm. One percent of the culture was transferred to 3 mL of fresh LB broth and grown at 37 °C and 180 rpm to OD_600_ 0.6, followed by protein induction with 100 μM of isopropyl β-D-thiogalactopyranoside (IPTG) and 250 μM of CuCl_2_. Expression of the protein was performed at 20 °C and 120 rpm for 4 h, followed by 16 h of static incubation. After the IPTG induction, 250 μL of the individual culture was transferred into a 96-well plate, and the OD_600_ value was measured using a Multiskan™ FC Microplate Photometer (ThermoFisher Scientific). The 96-well plate was centrifuged at 3500×*g* for 10 min using a centrifuge Z400 (HERMLE Labortechnik GmbH, Wehingen, Germany) to obtain cells for the resting cell assay. Next, the cell pellets were resuspended with 250 μL of 50 mM citrate phosphate buffer (pH 8.0) supplemented with 1 mM CuCl_2_. The resting cell assay was performed by adding 0.5 mM of 2,6-DMP as a substrate into each well and incubating at 70 °C for 2 min to compare laccase activity among mutants. A control experiment with *E. coli* strain BL21(DE3) harboring pECtLac was run simultaneously under the same assay conditions. The mutants with enhanced laccase activity were subjected to nucleotide sequencing at Macrogen Inc. (Seoul, South Korea) to confirm changed amino acids. Finally, the best mutant was selected for further site-directed mutagenesis experiments.

### Site-directed mutagenesis

Site-directed mutagenesis was performed at the V243 position of wild-type CtLac using Q5® Site-Directed Mutagenesis Kit according to the manufacturer’s protocol (New England BioLabs). Substitutions of V243 were introduced using the primer sets listed in Supplemental Table S1. The PCR conditions were followed by initial denaturation (98 °C for 30 s), 25 cycles of annealing (98 °C for 10 s, 59—65 °C for 15 s, and 72 °C for 3 min 30 s), and final extension (72 °C for 10 min). The resulting PCR products were treated with kinase, ligase, and *Dpn*I at room temperature for 15 min. *E. coli* strain DH5α was transformed with the resulting plasmids. Nineteen pECtLac plasmids substituted with the desired amino acids at the V243 position were obtained through nucleotide sequencing and their laccase activities were compared through spectrophotometric assay and oxygen consumption measurement.

### Expression and purification of enzymes

The 19 pECtLac mutants were separately cloned in *E. coli* strain BL21(DE3) for protein expression. The *E. coli* strain BL21(DE3) harboring plasmid with each CtLac V243 mutant was grown, and protein expression conducted as described above. After the protein induction, cells were harvested by centrifuge at 10,000×*g* for 15 min and washed twice with minimal salts basal medium for protein purification. Cell lysis was conducted using BugBuster™ protein extraction reagent (Sigma-Aldrich) according to the manufacturer’s protocol. The lysate was cleared by centrifugation and filtration using a 0.2-μm membrane filter (Advantec MFS Inc., Dublin, CA, USA) and used as a crude laccase enzyme source or further purified to homogeneity using HisTrap affinity chromatography. The lysate was applied in a His-Trap HP column (GE Healthcare, Chicago, IL, USA) and eluted with a linear gradient of imidazole from 0 to 250 mM buffered 20 mM Tris–HCl (pH 8.0). A fraction containing the laccase was desalted using a desalting column (GE Healthcare) and then concentrated using an Ultra Centrifugal Filter (Amicon, Miami, FL) to obtain concentrated high-purity protein for further experiments. The protein concentration was determined by a BCA protein assay kit (Pierce Biotechnology, Waltham, MA).

### Laccase activity assay

#### Spectrophotometric assay

The laccase activity of CtLac V243 mutants was determined by measuring the absorbance at 468 nm of the oxidized product from 2,6-DMP (Edens et al. 1999). The crude enzyme of all CtLac V243 mutants from the site-directed mutation was reacted with 0.5 mM 2,6-DMP in 50 mM citrate phosphate buffer containing 1 mM CuCl_2_ (pH 8.0) at 25 °C to compare laccase activities among the mutants. The concentration of the oxidized product of 2,6-DMP was calculated according to the Beer-Lambert law by considering the absorbance at 468 nm, the molar extinction coefficient (ε = 49.6 mM^−1^ cm^−1^), and path length (Islam and Harnett 2019). The results were used to select the mutant with the highest laccase activity, which was compared with wild-type CtLac using the purified protein.

#### Real-time oxygen measurement

The laccase activity of CtLac V243 mutants was determined by measuring oxygen consumption during the oxidation of 2,6-DMP at 25 °C. The real-time dissolved oxygen concentration in the reaction mixture was measured using an OXY-1 ST trace transmitter with a needle-type NHT-PSt7 oxygen microsensor (PreSens-Precision Sensing GmbH, Regensburg, Germany). The reaction mixture was 5 mL of 50 mM citrate phosphate buffer containing 0.5 mM 2,6-DMP and 1 mM CuCl_2_ (pH 8.0) in 15 mL serum bottle sealed with Teflon-lined rubber septa and aluminum caps. The needle-type optical sensor was dipped in the reaction mixture by penetrating the septa and the dissolved oxygen was measured in real-time during the whole reaction. The initial dissolved oxygen concentration of the reaction mixture was equilibrated by waiting for a few minutes after injecting the needle-type sensor. Once the real-time dissolved concentration was stable, crude or purified enzyme was added to the reaction mixture, which was continuously stirred on a magnetic plate to promote enzyme activity.

#### Biochemical characterization of V243D and V243M

The effects of different temperatures (20–90 °C) and pH (3.0–8.0) on the laccase activity of the purified V243D and V243M were compared with that for wild-type CtLac. The kinetic parameters (*K*_m_, *k*_cat_, and *k*_cat_/*K*_m_) of purified V243D and V243M were determined using different concentrations of 2,6-DMP (100–1000 μM) through the spectrophotometric assay and the real-time oxygen consumption measurement at 25 °C. These parameters were compared with those of purified wild-type CtLac. In addition, the kinetic study of the enzymes at 70 °C was performed based on the spectrophotometric assay. The data were analyzed using GraphPad Prism (v. 9; GraphPad, San Diego, CA) with the Michaelis–Menten equation for nonlinear regression.

#### Oxidative degradation of lignin model compound GGGE and rice straw using wild-type CtLac, V243D, and V243M

To compare the enzyme activity between wild-type CtLac and the selected CtLac V243 mutants on the degradation of the lignin model compound, each purified enzyme was treated with GGGE. Ten micrograms of the purified enzyme was reacted with 0.5 mM GGGE in 50 mM citrate phosphate buffer containing 1 mM CuCl_2_ (pH 8.0) at 70 °C and 120 rpm for 12 h. The reaction mixture was extracted with five volumes of ethyl acetate to analyze the residual GGGE and its product by its laccase activity. The organic phase was evaporated using a SpeedVac vacuum concentrator (Thermo Fisher Scientific). The residue was dissolved in high-purity grade methanol and followed by filtering with polyvinylidene fluoride (PVDF) syringe filter (Whatman-GE Healthcares, Pittsburgh, PA) before ultra-performance liquid chromatography-quadrupole-time-of-flight-tandem mass spectrometry (UPLC-Q-TOF–MS/MS) analysis (Impact II Q-TOF system, Bruker, Billerica, MA).

Rice straw was selected as the representative lignocellulosic biomass because it is an abundant byproduct in the agricultural environment. The preparation of rice straw was conducted following the protocol from our previous study (Yang et al. 2019). Briefly, rice straw collected from a local farm was rinsed with tap water to remove organic matter, such as soil, and then cut into 5 cm lengths and milled into pieces with an approximately 4 mm size. After repeated autoclaving at 121 °C for 1 h and washing with sterile deionized water, the rice straw was dried in an oven at 70 °C for 2 days. Ten micrograms of the purified enzyme was reacted with 2% (w/v) of the rice straw in 20 mL of 50 mM citrate phosphate buffer containing 1 mM CuCl_2_ (pH 8.0) at 70 °C for 6 h. At 3 and 6 h of reaction, the sample was taken and extracted with five volumes of ethyl acetate to extract the lignin monomers released from the rice straw by the enzyme treatment. The organic phase was evaporated, and the residue was dissolved in high-purity 40% aqueous acetonitrile, followed by filtering with a PVDF syringe filter before the UPLC-Q-TOF–MS analysis.

#### Analytical methods

After the enzyme treatment, the products from GGGE or rice straw were analyzed using UPLC-Q-TOF–MS/MS. UPLC was equipped with a photodiode array detector and a reverse-phase C18 column (Bruker, Intensity Solo 2 C18, 100 × 2.0 mm, 2.0 μm in particle size). The mobile phase was composed of deionized water containing 0.1% formic acid (solvent A) and acetonitrile containing 0.1% formic acid (solvent B). UPLC was operated in a linear gradient for solvent B 10–55% for 0–15 min, 55–100% for 15–17 min, and 100% for 17–20 min, with subsequent equilibrium at 10% for 5 min. The column temperature was 40 °C, and the injection volume was 3 μL. The MS conditions were as follows: negative ionization mode; mass range, *m/z* 50—1500; capillary voltage, 4500 V; nebulizer, 1.2 bar; dry gas flow rate, 10.0 L/min; and dry temperature, 200 °C. For collision-induced dissociation (CID)-MS/MS, the collision energy was set to 5–15 eV.

#### Identification of oxidative products from GGGE and lignin-derived monomers from rice straw

The oxidative product from GGGE after treatment of CtLac wild-type and CtLac V243 mutants was identified based on the CID fragmentation patterns from LC–MS/MS analysis (Ghatge et al. 2018). Briefly, the typical neutral loss (*m/z*) in the fragments could be from functional groups of phenolic compounds, such as methyl radical and formaldehyde. Similarly, the identification of lignin-derived monomers by the enzyme treatments was conducted based on the UV-HPLC–MS/MS-based method developed in our previous study (Yang et al. 2019). The quantification of the lignin-derived monomers in the reaction mixture was performed by calculating the peak area of extracted ion chromatogram (EIC) for each compound using the calibration curve of authentic vanillin from an EIC.

## Results

### Construction and screening of CtLac mutants with enhanced laccase activity from random mutagenesis

A random mutagenesis library was constructed by error-prone PCR using the GeneMorph II EZClone domain mutagenesis kit. The reaction conditions were set to introduce 0–4.5 mutations per 1000 bps of CtLac coding gene. A total of 1300 transformants were picked randomly, and their laccase activities were examined using a resting cell assay in 96-well plates. Among the tested transformants, one transformant named RM484 showed a 30% increase in laccase activity more than in wild-type CtLac and was selected for further experiments. The RM484 sequence has two mutations at V243D and I468V among 516 amino acids of wild-type CtLac. Considering that isoleucine and valine are categorized into the same amino acid group due to their similar structures with a hydrophobic side chain, I468V was not considered a significant mutation point affecting the enzyme activity. Therefore, valine at the 243 position was assumed to be an essential influential factor in the enhanced laccase activity. Finally, it was selected as the target amino acid for site-directed mutagenesis.

### Comparison of laccase activity among CtLac V243 mutants from site-directed mutagenesis based on the spectrophotometric assay and the real-time oxygen measurement at 25 °C

**Fig. 1:**
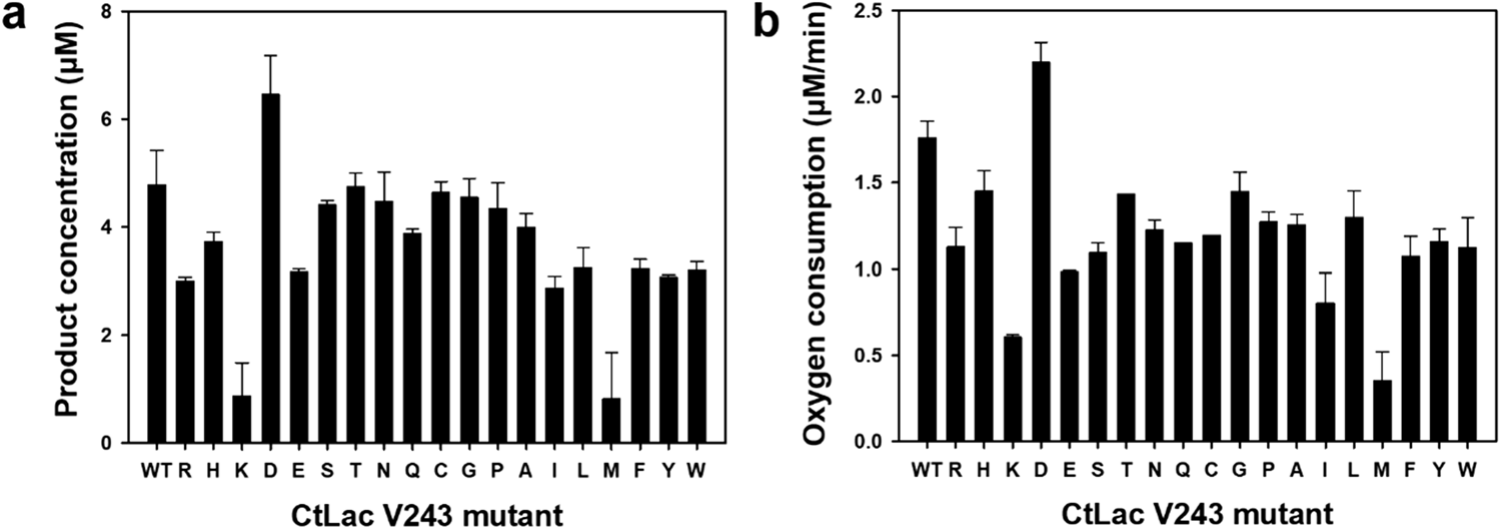
Comparison of laccase activity of cell-free extract of wild-type CtLac (WT) and CtLac V243 mutants in 50 mM citrate phosphate buffer (pH 8.0) containing 0.5 mM 2,6-DMP at 25 °C and pH 8.0 using **a** the spectrophotometric assay and **b** the real-time oxygen measurement. Each amino acid letter abbreviation indicates a substitution mutation at V243 position of wild-type CtLac. Values are mean of triplicate determinations ± standard deviation

pECtLac was used as a template to obtain substitutions at the V243 position. Nineteen mutants were consequently obtained from the site-directed mutagenesis. The mutants’ laccase activity was compared based on the results of the spectrophotometric assay and real-time oxygen measurement during the enzyme reaction at 25 °C and pH 8.0. The overall trends for increase or decrease in the product concentration and oxygen consumption by the CtLac V243 mutants were similar in both measurements (Fig. 1). The concentration of the oxidized product from 2,6-DMP by wild-type CtLac was 4.8 μM at 25 °C and pH 8.0. (Fig. 1a). The substitution of V243 by aspartic acid (D) showed 35% enhanced enzyme activity than wild-type CtLac under the spectrophotometric assay. The other substitutions showed varied enzyme activities. Specifically, six substitutions, including serine (S), threonine (T), asparagine (N), cysteine (C), glycine (G), and proline (P), showed about a 10% decrease in enzyme activity than wild-type CtLac under the spectrophotometric assay (Fig. 1a). In addition, 10 substituents including arginine (R), histidine (H), glutamic acid (E), glutamine (Q), alanine (A), isoleucine (I), leucine (L), phenylalanine (F), tyrosine (Y), and tryptophan (W) showed about a 20–40% decrease in enzyme activity than wild-type CtLac. Remarkably, substitutions of V243 by lysine (K) or methionine (M) showed highly decreased laccase activity (18.3% and 17.0%, respectively) compared to wild-type CtLac.

Oxygen consumption rate (μM O_2_/min) was evaluated by the real-time measurement of the dissolved oxygen in the reaction mixture during the enzyme reaction. Each enzyme was added to the reaction mixture after stabilizing the dissolved oxygen value for 3 min at around 295–310 μM. A bumping line was observed while the needle-type optical oxygen sensor was dipping into the reaction mixture through Teflon-lined rubber septa, which might be from the insertion of oxygen in the enzyme solution into the reaction mixture. This bumping line was commonly observed in all reactions within 5 μM; therefore, it was considered to be the initial dissolved oxygen concentration. Only V243D showed 25% higher enzyme activity among the mutants than wild-type CtLac (Fig. 1b).

### Comparison of biochemical characterization of purified V243D and V243M with wild-type CtLac based on the spectrophotometric assay

**Fig. 2:**
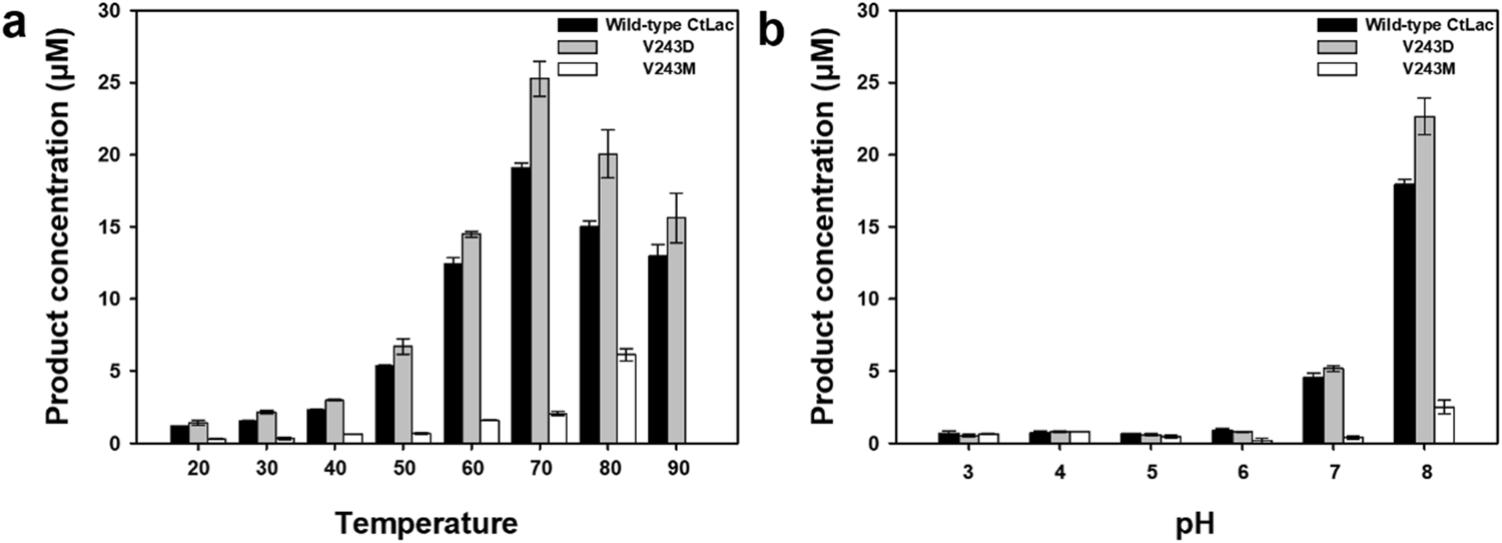
Comparison of laccase activity of purified wild-type CtLac (WT), V243D, and V243M in the presence of 0.5 mM 2,6-DMP at different **a** temperatures at pH 8.0 and **b** pH at 70 °C. Values are mean of triplicate determinations ± standard deviation

Among the 19 V243 mutants, V243D was selected as the best mutant because it had the highest laccase activity based on the results of both the spectrophotometric assay and real-time oxygen measurement (Fig. 1). Therefore, its biochemical characterization was compared with wild-type CtLac and a V243M that had showed the least laccase activity. The biochemical characterization of the V243D and V243M was conducted based on only the results of the spectrophotometric assay because the needle-type optical fiber oxygen sensor is limited by temperature ranges up to 50 °C. V243D enhanced laccase activity 16–42% more than wild-type CtLac in the tested temperature ranges (20 °C to 90 °C) (Fig. 2a). However, V243D laccase activity was not affected significantly by the change in pH, except for pH 8.0 (Fig. 2b). In particular, V243D showed the most enhanced laccase activity at 70 °C and pH 8.0 by producing 25.3 μM of the oxidized product of 2,6-DMP, while wild-type CtLac produced 19.1 μM. Thus, we assumed that the condition of 70 °C and pH 8.0 is the optimal reaction condition for the V243D and wild-type CtLac. However, V243M showed about an 80–90% decrease in enzyme activity compared to wild-type CtLac, even at the optimal enzyme conditions (i.e., 70 °C and pH 8.0).

### Comparison of kinetic parameters of V243D and V243M with wild-type CtLac based on the spectrophotometric assay and the real-time oxygen consumption measurement at 25 °C and pH 8.0

The kinetic parameters of V243D and V243M were investigated based on the results of the spectrophotometric assay and real-time oxygen consumption measurement using different concentrations of 2,6-DMP. The data were analyzed using the Michaelis–Menten equation by nonlinear regression and compared with wild-type CtLac. The results of the spectrophotometric assay showed that V243D exhibited *K*_m_ and *k*_cat_ values of 137.2 μM and 2.7 s^−1^ at 25 °C and pH 8.0, respectively, which was 12.7% lower and 145.5% higher than those of wild-type CtLac, respectively (Table 1a). The *k*_cat_/*K*_m_ value of V243D was determined to be 0.02 s^−1^ μM^−1^, which was about 2.9-fold larger in catalytic efficiency than wild-type CtLac. In particular, the specific activity of 10 μg purified V243D in the presence of 500 μM 2,6-DMP at 25 °C and pH 8.0 was 347.7 ± 16.2 μmol/min/mg, which was 2.3-fold higher than that of wild-type CtLac. However, V243M showed *K*_m_ and *k*_cat_ values of 674.2 μM and 0.1 s^−1^ at 25 °C and pH 8.0, respectively, which were 329.0% higher and 91.0% lower than the related values for wild-type CtLac.

**Table 1:** Kinetic parameters of wild-type CtLac (WT), V243D, and V243M for the oxidation of different concentrations of 2,6-DMP ranging from 100 to 1000 μM using **a** the spectrophotometric assay and **b** the oxygen consumption measurement at 25 °C and pH 8.0

a	Construct	*K*_m_ (μM)	*k*_cat_ (s^−1^)	*k*_cat_/*K*_m_ (s^−1^ μM^−1^)
	WT	157.2	1.1	0.007
	V243D	137.2	2.7	0.020
	V243M	674.2	0.1	0.0001
b	Construct	*K*_m(O2)_ (μM)	*k*_cat(O2)_ (s^−1^)	*k*_cat(O2)_/*K*_m(O2)_ (s^−1^ μM^−1^)
	WT	497.0	3.5	0.007
	V243D	332.3	4.2	0.013
	V243M	87.7	0.7	0.007

**Fig. 3:**
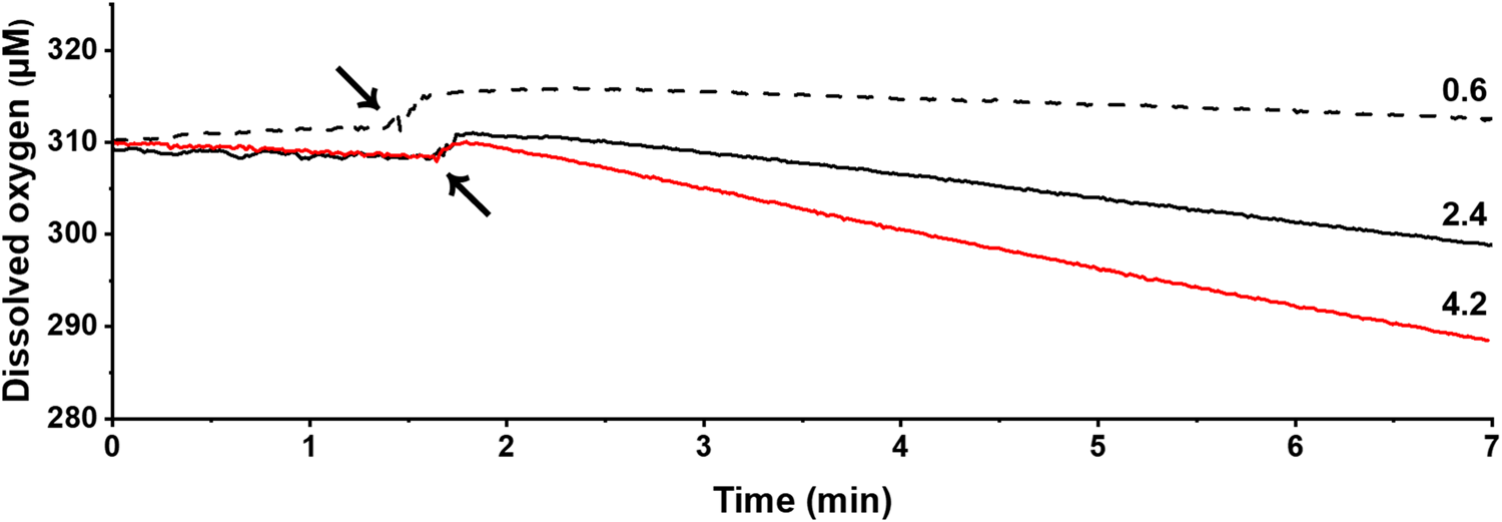
Real-time measurement of dissolved oxygen consumption by purified wild-type CtLac, V243D, and V243M in the presence of 500 μM of 2,6-DMP at 25 °C and pH 8.0. Black arrows indicate the time of each enzyme addition. The figures on the right side mean the oxygen consumption rate (μM O_2_/min)

The kinetic parameters of the mutants and wild-type CtLac were also obtained from the oxygen consumption measurement (Table 1b). The oxygen consumption rate (μM O_2_/min) in the reaction mixture containing different concentrations of 2,6-DMP was measured in real time as shown in Supplemental Table S2. V243D exhibited *K*_m(O2)_ and *k*_cat(O2)_ values of 332.3 μM and 4.2 s^−1^, respectively, which were 33.1% lower and 20.0% higher than the respective values for wild-type CtLac at 25 °C and pH 8.0 (Table 1b). However, V243M showed *K*_m(O2)_ and *k*_cat(O2)_ of 87.7 μM and 0.7 s^−1^ at 25 °C and pH 8.0, respectively, which was 82.4% lower and 80.0% lower that the respective values for wild-type CtLac. There was a distinct difference between the oxygen consumption rate (μM O_2_/min) among wild-type CtLac, V243D, and V243M in the presence of 500 μM 2,6-DMP at 25 °C and pH 8.0, following the order V243D > wild-type CtLac > V243M at 4.2, 2.4, and 0.6, respectively (Fig. 3).

### Comparison of kinetic parameters of V243D and V243M mutants with wild-type CtLac based on the spectrophotometric assay under the optimal enzyme conditions at 70 °C and pH 8.0

As investigated in our previous study, CtLac showed the highest laccase activity at a high temperature of 70 °C and pH 8.0 because it originated from thermoalkaliphilic bacterium, namely, *C. thermarum* strain TA2.A1 (Ghatge et al. 2018). In other words, the absolute concentrations of the oxidized product from wild-type CtLac and V243 mutants were substantially higher at 70 °C than those at 25 °C (Supplemental Fig. S1 and Fig. 1b). Therefore, the kinetic parameters of V243D and V243M were investigated at 70 °C and pH 8.0 to compare with the results from wild-type CtLac. V243D showed *K*_m_ and *k*_cat_ values of 127.3 μM and 15.3 s^−1^, respectively, which were 13.6% lower and 19.5% higher than for wild-type CtLac, respectively (Table 2). Moreover, the *k*_cat_/*K*_m_ value of V243D was 0.12 s^−1^ μM^−1^, which showed 1.4-fold more catalysis efficiency compared to wild-type CtLac. Moreover, the specific activity of 10 μg purified enzyme in the presence of 500 μM 2,6-DMP at 70 °C and pH 8.0 was 2141.8 μmol/min/mg, which was onefold higher than that of wild-type CtLac. However, V243M showed *K*_m_ and *k*_cat_ values of 1127 μM and 1.5 s^−1^ at 70 °C and pH 8.0, respectively, which were 665.1% higher and 88.3% lower than the respective values for wild-type CtLac.

**Table 2:** Kinetic parameters of wild-type CtLac (WT), V243D, and V243M for the oxidation of different concentrations of 2,6-DMP ranging from 100 to1000 μM using the spectrophotometric assay at 70 °C and pH 8.0

Construct	*K*_m_ (μM)	*k*_cat_ (s^−1^)	*k*_cat_/*K*_m_ (s^−1^ μM^−1^)
WT	147.3	12.8	0.087
V243D	127.3	15.3	0.120
V243M	1127	1.5	0.0013

In the present study, we performed random and site-directed mutagenesis and compared enzyme activity using the spectrophotometric assay and real-time oxygen measurement to select the best mutant with enhanced enzyme activity, although there were no references in the protein structures. Therefore, this atypical trial could be applied as a more powerful tool to evaluate and broaden the range of novel oxidase enzymes.

### Enhancement of oxidative degradation of lignin model compound and rice straw using V243D at 70 °C and pH 8.0

**Fig. 4:**
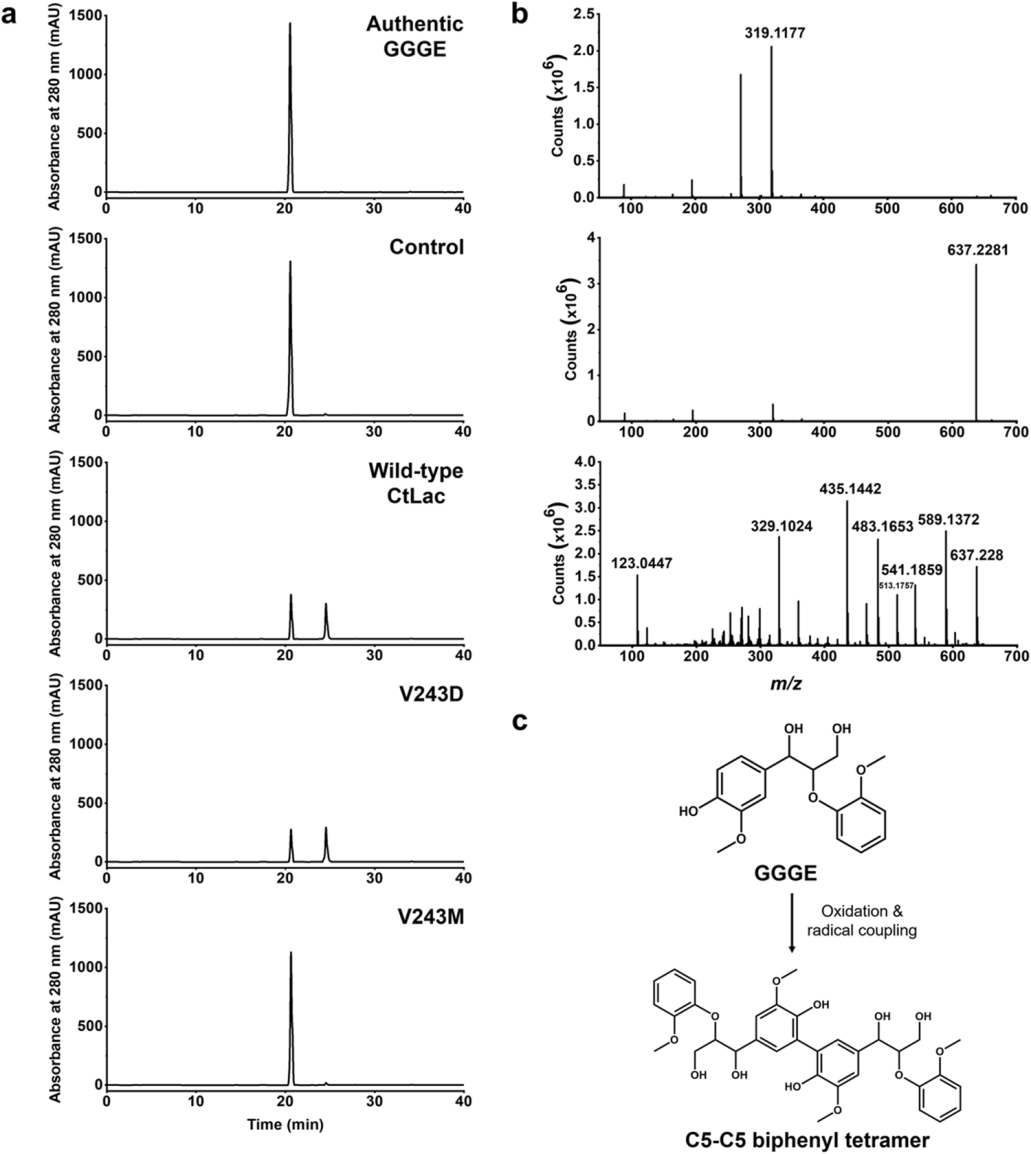
UPLC-Q-TOF–MS/MS analysis of oxidation of dimeric lignin model compound, GGGE using purified wild-type CtLac, V243D, and V243M in 50 mM citrate phosphate buffer (pH 8.0) at 70 °C. **a** LC chromatograms of authentic GGGE, control without enzymes, wild-type CtLac, V243D, and V243M, sequentially, **b** MS spectra of authentic GGGE and V243D-catalyzed oxidation product of GGGE, and its MS/MS spectrum, sequentially, **c** oxidized form of product from GGGE by V243D

The enhancement of lignin degradation by V243D was determined using GGGE and rice straw. Purified V243D and V243M were treated on each substrate at 70 °C and pH 8.0, and the enzymatic activity on the oxidative lignin degradation was compared with that of wild-type CtLac. After 6 h of the enzymatic treatment, 10% higher GGGE oxidation was observed in V243D than wild-type CtLac, while V243M slightly oxidized GGGE (Fig. 4a). The *m/z* value of the product was 637.2 of deprotonated ion in the negative ionization mode [M-H]^−^, which was expected as a dimer form of GGGE (Fig. 4b). In addition, the CID fragmentation patterns of the product from GGGE by V243D treatment exactly matched the results of wild-type CtLac in our previous study (Ghatge et al. 2018). Briefly, there were typical neutral losses of 15 Da (CH_3_•, methyl radical), 18 Da (H_2_O, water), 30 Da (CH_2_O, formaldehyde), 48 Da (H_2_O + CH_2_O), and 124 Da (C_7_H_8_O_2_, guaiacol) among well-known characteristic fragment ions from β-aryl ether linkage, which comprises > 50% of the interunit linkages found in all types of lignin (Morreel et al. 2010; Zakzeski et al. 2010). The *m/z* values of major fragment ions were 589.1, 541.1, 483.1, 435.1, and 329.1, which indicated [M-H-H_2_O-CH_2_O]^−^, [M-H-2H_2_O-2CH_2_O]^−^, [M-H-guaiacol-CH_2_O]^−^, and [M-H-2guaiacol-CH_2_O]^−^, respectively (Fig. 4b). These *m/z* fragment patterns exactly matched our previous results from CtLac treatment on GGGE (Ghatge et al. 2018). In other words, GGGE might be transformed to C_5_-C_5_ biphenyl tetramer by the initial enzymatic oxidation and radical–radical coupling (Fig. 4c). Considering that V243D has lower *K*_m_ and higher *k*_cat_ values than wild-type CtLac, it might enhance oxidation activity on GGGE better than wild-type CtLac.

**Fig. 5:**
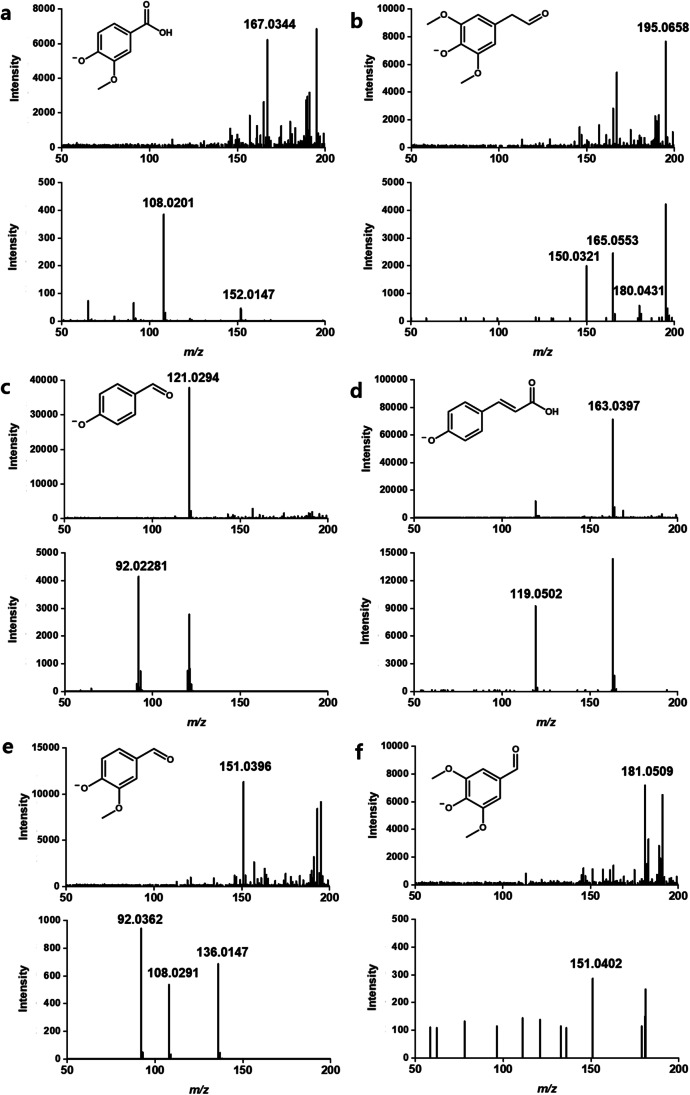
UPLC-Q-TOF–MS (top) and MS/MS (down) spectra of the lignin-derived monomers from rice straw after treatment of purified V243D. **a** Vanillic acid, **b** homosyringaldehyde, **c** *p*-hydroxybenzaldehyde, **d** *p*-coumaric acid, **e** vanillin, and **f** syringaldehyde

Furthermore, V243D and V243M were treated on rice straw as a representative lignocellulosic biomass, and the production of lignin-derived monomers was compared with wild-type CtLac. The six lignin-derived monomers were detected at *m/z* values of (a) 167.03, (b) 195.06, (c) 121.02, (d) 163.03, (e) 151.03, and (f) 181.05, and identified as vanillic acid, homosyringaldehyde, *p*-hydroxybenzaldehyde, *p*-coumaric acid, vanillin, and syringaldehyde, respectively (Fig. 5). The lignin monomers were quantified using the peak area of EIC for the compounds, which was used to calculate the concentration of the compounds compared to the EIC for authentic vanillin (0–200 μM). The above six lignin-derived compounds commonly have a phenolic group that is deprotonated in negative ionization mode; therefore, the compounds’ concentrations could be calculated from this calibration curve, even in the absence of authentic chemicals, such as homosyringaldehyde.

Specifically, the concentrations of homosyringaldehyde, *p*-hydroxybenzaldehyde, and vanillin were 17.8 μM, 91.7 μM, and 98.0 μM, respectively, after 3 h of V243D treatment (Table 3). However, wild-type CtLac produced lower concentrations of compounds than V243D (i.e., 13.8 μM, 74.2 μM, and 90.2 μM, respectively). Significantly, vanillin was still increased at 6 h of enzyme treatment, even though homosyringaldehyde and *p*-hydroxybenzaldehyde were decreased due to possible employment as substrates in the laccase activity. In contrast, V243M produced similar concentrations of these benzaldehyde compounds from the control sample containing only rice straw without enzymes, which meant that V243M had no significant effect on the release of lignin monomers due to its low enzyme activity. The total concentration of lignin-derived monomer from rice straw was around 265 μM after V243D treatment, which was higher than wild-type CtLac. Consequently, V243D produced much more high-value benzaldehyde compounds from rice straw than did wild-type CtLac.

**Table 3:** Quantification of lignin-derived monomers from rice straw after treatment of purified wild-type CtLac (WT), V243D, and V243M

Compound	*m/z*	Concentration of lignin-derived monomers produced after enzyme treatment (μM)^a^							
		3 h				6 h			
		Control^b^	WT	V243D	V243M	Control	WT	V243D	V243M
Vanillic acid	167.03	10.4 ± 0.9	7.0 ± 0.0	7.5 ± 0.3	9.9 ± 0.2	11.0 ± 0.4	8.3 ± 0.6	9.0 ± 0.6	9.9 ± 0.3
Homosyringaldehyde	195.06	11.6 ± 1.4	13.8 ± 1.1	17.8 ± 0.3	11.6 ± 0.3	6.8 ± 0.9	8.2 ± 0.5	8.8 ± 0.3	6.9 ± 0.7
*p*-Hydroxybenzaldehyde	121.02	61.1 ± 1.4	74.2 ± 5.3	91.7 ± 1.0	59.0 ± 4.5	46.5 ± 1.0	56.2 ± 0.0	60.3 ± 3.8	47.5 ± 3.4
*p*-Coumaric acid	163.03	89.7 ± 6.4	90.2 ± 7.2	98.0 ± 3.6	95.0 ± 2.1	135.2 ± 1.5	121.0 ± 7.5	134.4 ± 1.3	141.9 ± 4.0
Vanillin	151.03	10.3 ± 0.3	20.7 ± 0.0	21.0 ± 0.3	10.2 ± 0.8	10.8 ± 2.9	23.4 ± 1.0	25.0 ± 0.6	11.2 ± 0.4
Syringaldehyde	181.05	30.9 ± 2.4	28.4 ± 0.4	29.3 ± 0.5	29.0 ± 0.4	25.7 ± 2.8	28.2 ± 1.2	27.6 ± 0.9	28.7 ± 1.2
Total concentration of lignin- derived monomer (μM)^c^		214 ± 2.8	243.3 ± 2.5	265.4 ± 4.9	214.7 ± 5.2	235.9 ± 0.1	245.4 ± 7.6	265.1 ± 4.6	246.1 ± 0.2

^a^Concentration was calculated from EIC peak areas based on the calibration curves using authentic vanillin

^b^Control sample contained only rice straw without enzymes and reacted under the same experimental conditions with enzyme treatments

^c^Total concentration of lignin-derived monomers from rice straw after the treatments (μM)

## Discussion

The comparison of laccase activity among CtLac V243 mutants using the spectrophotometric assay and real-time oxygen measurement found that the results of the spectrophotometric assay considerably overestimated the laccase activity. For example, six substitutions, including S, T, N, C, G, and P, exhibited only a 10% decrease in enzyme activity than wild-type CtLac from the spectrophotometric assay. However, the real-time oxygen measurement showed 18–38% decrease in enzyme activity, which indicates that the spectrophotometric assay has some limitations in evaluating oxidase enzyme activity, even though the assay method is simple and easy to perform. The resulting values from the spectrophotometric assay could have been interfered from various factors, such as the limit of detection of substrates or products, a need for absorbance at a particular wavelength, and unintended side products of enzyme reactions (Demarche et al. 2015).

During laccase catalysis, 2,6-DMP undergoes single-electron oxidation and transforms to 2,6-dimethoxy-phenoxy radical species. Once the unpaired electron resonates with *p*-radical species in the benzene ring, the consecutive dimeric or polymeric reaction will be undergone nonspecifically. Several dimeric compounds have been reported as results from 2,6-dimethoxy-phenoxy radicals, such as 3,3′,5,5′-tetramethoxydiphenoquinone, 3,3′,5,5′-tetramethoxybiphenyl-4,4′-diol, and 4-(2,6-dimethoxy-phenoxy)-2,6-dimethoxyphenol from 2,6-DMP by laccase-catalyzed reaction (Kallio et al. 2009; Schirmann et al. 2018; Wan et al. 2008). In addition, polymeric oxidative products, such as C_1_-C_1_ biphenyl polymer or C_1_-O-C_1_ biphenyl ether polymers, could be produced from consecutive radical-driven reactions (Wan et al. 2008). However, the spectrophotometric assay generally detects only 3,3′,5,5′-tetramethoxydiphenoquinone because of the absorbance of quinone-based structure at 468 nm. In other words, the spectrophotometric assay absorbs all types of quinone-based products from the nonspecific reaction of radicals during the enzyme reaction, which could result in inconclusive findings from the evaluation and comparison of enzyme activity.

Furthermore, the spectrophotometric assay for measuring the oxidized products from laccase activity could be influenced by assay conditions, such as pH and reaction time. For example, phenolic compounds, such as 2,6-DMP and caffeic acid, increase auto-oxidation above pH 8.5, while nonphenolic compounds, such as 2,2′-azino-bis (3-ethylbenzothiazoline-6-sulfonate) (ABTS) and syringaldazine, exhibit no auto-oxidation at a pH range of 2.6–8.0 (Brander et al. 2014). Therefore, selecting optimal substrates is especially important for successfully determining laccase activity using the spectrophotometric assay.

Meanwhile, the measurement of oxygen consumption during the laccase reaction circumvents the drawbacks of the spectrophotometric assay. Laccase catalyzes the single-electron oxidation of substrate and transfers four electrons to one molecule of oxygen, which is reduced to two molecules of water. Therefore, comparing oxygen consumption might be an obvious criterion for evaluating enzyme activity. For example, the technique could be applied to develop a laccase-mediator-system (LMS) to improve the catalytic efficiency of the enzyme (Kirsch et al. 2015; Euring et al. 2022; Hilgers et al. 2018). On the other hand, Euring et al. (2022) evaluated the performance of different natural mediators in the enzymatic oxidation of lignin by comparing oxygen consumption rates in different LMS and found that the 2,6-DMP mediator significantly enhanced the oxidation of lignosulfonate and Indulin AT with a big decrease in oxygen saturation. On the other hand, the oxygen consumption of LMS with the help of ABTS was faster than that of 1-hydroxy-benzotriazole (HBT) supported-LMS in the oxidation of GGGE (Hilgers et al. 2018). More interestingly, these LMS resulted in different oxidation pathways. For example, HBT-LMS led to the extensive polymerization of GGGE; however, ABTS-LMS directly oxidized GGGE by both laccase and ABTS radical cations, which created a stable product instead of further polymerization. These results implied that the detection of the specific oxidized product by laccase reaction would not be a direct method for evaluating enzyme activity. Furthermore, it would be very hard to predict various polymerized products in cases of complex substrates like lignin.

The measurement of oxygen consumption during the laccase reaction could be a more-accurate method to evaluate and compare the enzyme activity among mutants than the spectrophotometric assay, especially in mutagenesis research. Moreover, oxygen consumption measurements could be applied in other oxidases and oxygenase enzymes.

Considering the results from the screening of mutants with enhanced laccase activity based on the spectrophotometric assay and real-time oxygen consumption measurement, two mutants (i.e., V243K and V243M) showed significantly decreased laccase activity compared to wild-type CtLac (Fig. 1). In the case of V243K, the substitution of Val to Lys might reduce the solubility of the protein because an inclusion body was observed in the cell lysis step of the crude laccase enzyme preparation (Supplemental Fig. S2). This result might infer that V243K could not induce the appropriate conformational change of the enzyme, resulting in the significant loss of enzyme activity.

Meanwhile, the decrease in the laccase activity of V243M might be from the copper-binding issue with the T1 copper-binding site of laccase or the vicinity of the catalytic coppers. This finding could be deduced from the color difference in the *E. coli* (pECtLac-V243M) cell pellet. Specifically, the color of the *E. coli* (pECtLac) and *E. coli* (pECtLac-V243D) cell pellets turned greenish during the protein overexpression in the presence of CuCl_2_. However, the color of the *E. coli* (pECtLac-V243M) cell pellet remained yellowish (Supplemental Fig. S3). This green color might be a result of the coordination of Cu^2+^ in the T1 site of laccase, which is the characteristic adsorption of the oxidized Cu^2+^ state at 600 nm (Christopher et al. 2014). Therefore, the substitution of Val to Met could affect the copper-binding activity of wild-type CtLac. In other words, V243D might induce favorable conformational changes in the enzyme’s active site better than wild-type CtLac because the catalytic efficiency of laccase is generally proportional to the redox potential of T1 copper. Although this deduction might be imperfect without the exact protein structure of wild-type CtLac, it could consider Val at the 243 position of CtLac to be one of the key amino acids contributing to the catalytic efficiency of the enzyme because of the noticeable differences among the mutants. Although it is necessary to predict the critical residues of CtLac using the protein structure data of other laccases available from the Protein Data Bank archive (https://www.rcsb.org/), we found a low conservation of amino acid sequence (< 30%) compared with the previously known structure for bacterial laccase (Xie et al. 2018). Therefore, further studies based on the protein structural data of wild-type CtLac and mutants are needed to prove the above assumption.

The mutation at the V243 position of wild-type CtLac affected laccase activity by resulting in changes in the kinetic parameters. In particular, V243D showed improved enzyme activity with decreased *K*_m_ and increased *k*_cat_. Thus, our results could provide a pathway for improving the catalytic activity of CtLac by investigating the molecular structure further. Indeed, most investigations on mutagenesis to enhance enzyme activity have been performed between well-known laccases with high amino acid identities, such as *Bacillus licheniformis* and *Bacillus subtilis* (Bu et al. 2020; Mollania et al. 2011; Wang et al. 2017).

The action of CtLac on rice straw can be assumed to differ from that on simple phenolic compounds, such as 2,6-DMP and GGGE. CtLac could oxidize 2,6-DMP by directly abstracting a single-electron from this phenolic substrate, resulting in phenoxyl radicals and sequential radical coupling. However, CtLac could not directly abstract electrons from the nonphenolic group of lignin due to its intrinsically lower redox potential of laccase than in the nonphenolic group (Couto and Herrera 2006). As proposed in our previous study, this characteristic can be overcome by the action of lignin-derived monomers from rice straw as natural mediators in the CtLac-driven biodegradation of terminal β-*O*-4 type bond in rice straw (Yang et al. 2019). These mediators oxidized by CtLac might abstract electrons from the C_1_ carbon of β-*O*-4 type lignin. In addition, the C_1_ radical-driven cleavage of C_α_-C_β_ bond produced benzaldehydes, such as vanillin. Based on this proposed mechanism, V243D could oxidize a larger amount of lignin-derived monomers than wild-type CtLac, resulting in much-oxidized mediators with faster oxygen consumption during the enzyme treatment. Consequently, V243D could foster the production of much higher value benzaldehyde chemicals, such as *p*-hydroxybenzaldehyde and vanillin, than wild-type CtLac.

In the present study, we performed a two-step mutagenesis involving random mutagenesis followed by site-directed mutagenesis to obtain an engineered CtLac mutant with enhanced catalytic efficiency. Two methods were employed to compare the laccase activity among the mutants: namely, the spectrophotometric assay and real-time oxygen measurement. V243D showed a 35% enhanced laccase activity than wild-type CtLac under the spectrophotometric assay. However, V243D exhibited only 25% higher enzyme activity than wild-type CtLac according to the oxygen consumption measurement. Therefore, the real-time measurement of oxygen consumption could be a more-accurate method for evaluating oxidase activity in mutagenesis research, considering the limitations of the spectrophotometric assay. V243D showed higher catalytic efficiency with more decreased *K*_m_ and increased *k*_cat_ values compared to wild-type CtLac. Moreover, the results showed the enhanced oxidative degradation of lignin under the optimal enzymatic conditions (i.e., 70 °C and pH 8.0) with 10% higher GGGE oxidation and a 5–30% increase in high-value aldehyde production than wild-type CtLac. Therefore, we could consider Val at the 243 position of CtLac as one of the critical amino acids contributing to the catalytic efficiency of the enzyme, although the protein structure information of CtLac is absent.

## Supplementary Information

Below is the link to the electronic supplementary material.

Supplementary file1 (PDF 526 kb)

## Data Availability

All data generated or analyzed during this study are included in this published article.
