# Supplementary material for: Improvement of thermoalkaliphilic laccase (CtLac) by a directed evolution and application to lignin degradation
Source: Appl Microbiol Biotechnol. 2022 Dec 7;107(1):273–86. doi: 10.1007/s00253-022-12311-4 (PMC9750922; doi:10.1007/s00253-022-12311-4)
Supplement: Supplementary file 1 — Supplementary file1 (PDF 526 kb) [file 253_2022_12311_MOESM1_ESM.pdf]

Supplementary Information

Improvement of Thermoalkaliphilic Laccase (CtLac) by A Directed Evolution and Application to Lignin Degradation

Youri Yang[a], Sunil Ghatge[a,b], and Hor-Gil Hur[a]*

[a]School of Earth Sciences and Environmental Engineering, Gwangju Institute of Science and Technology (GIST), Gwangju 61005, Republic of Korea

[b]GREEN BIO Co. Ltd, Gwangju 61005, Republic of Korea

*Corresponding author

Professor. Hor-Gil Hur

E-mail: hghur@gist.ac.kr, Tel: +82-62-715-2437, Fax: +82-62-715-2434.

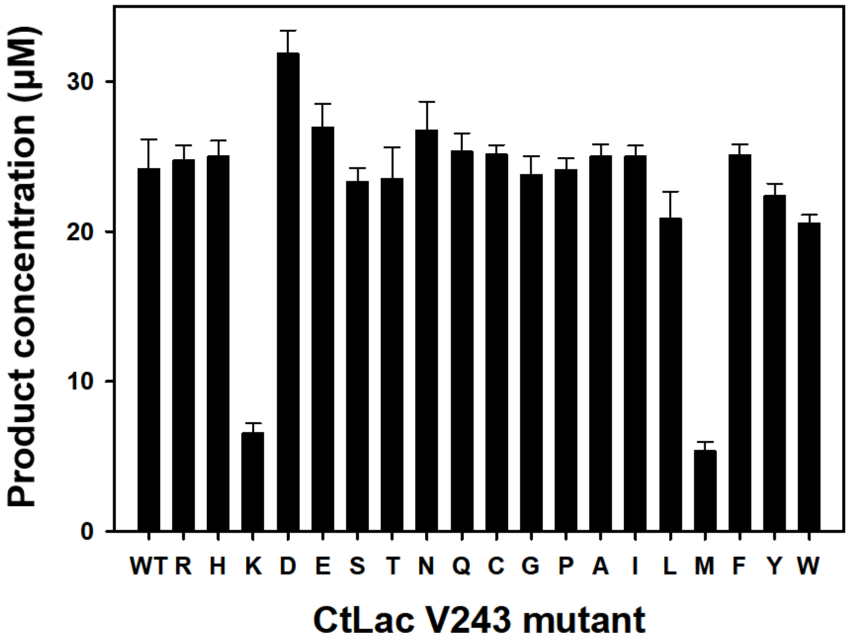

Fig. S1 Comparison of laccase activity of cell-free extract of wild-type CtLac (WT) and CtLac V243 mutants in 50 mM citrate phosphate buffer (pH 8.0) containing 0.5 mM 2,6-DMP at 70℃ and pH 8.0 using the spectrophotometric assay. Each amino acid letter abbreviation indicates a substitution mutation at V243 position of wild-type CtLac. Values are mean of triplicate determinations ± standard deviation.

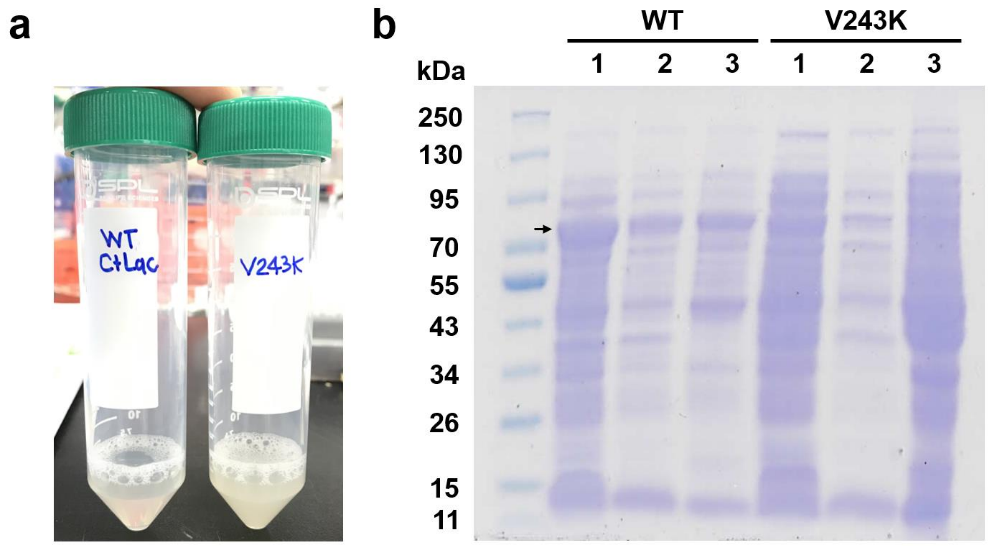

Fig. S2 Formation of inclusion body of *E. coli* (pECtLac-V243K). (a) Cell-free extract and (b) SDS-PAGE analysis of *E. coli* (pECtLac-V243K) after the protein overexpression. (1) whole-cell proteins, (2) soluble proteins, and (3) insoluble proteins. An arrow indicates CtLac. WT: wild-type CtLac.

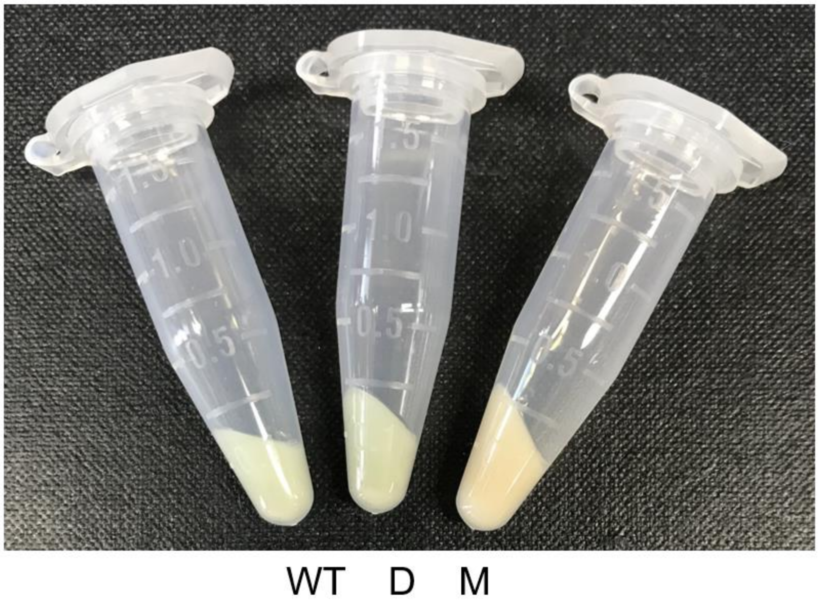

Fig. S3 Color differences among *E. coli* cell pellets harboring pECtLac (WT), pECtLac-V243D (D), and pECtLac-V243M (M) after the protein overexpression in the presence of $CuCl_2$.

Table S1 Primer sequences used in site-directed mutagenesis.

No.	Amino acid[a]	Primer name	Sequence (5' to 3')
1	Arginine (R)	V243R-F	AGCATCACCGCGTATCGAAGCCATTG
		V243R-R	TTTCCGTTCACAATTAACG
2	Histidine (H)	V243H-F	AGCATCACCGCATATCGAAGCCATTG
		V243H-R	TTTCCGTTCACAATTAACG
3	Lysine (K)	V243K-F	AGCATCACCGAAAATCGAAGCCATTG
		V243K-R	TTTCCGTTCACAATTAACG
4	Aspartic acid (D)	V243D-F	AGCATCACCGATATCGAAGCCA
		V243D-R	TTTCCGTTCACAATTAACGTATCATAC
5	Glutamic acid (E)	V243E-F	AGCATCACCGGAAATCGAAGCCA
		V243E-R	TTTCCGTTCACAATTAACGTATC
6	Serine (S)	V243S-F	AGCATCACCGAGCATCGAAGCCATTG
		V243S-R	TTTCCGTTCACAATTAACGTATC
7	Threonine (T)	V243T-F	AGCATCACCGACCATCGAAGCCATTG
		V243T-R	TTTCCGTTCACAATTAACGTATC
8	Asparagine (N)	V243N-F	AGCATCACCGAATATCGAAGCCATTG
		V243N-R	TTTCCGTTCACAATTAACGTATC
9	Glutamine (Q)	V243Q-F	AGCATCACCGCAGATCGAAGCCATTG
		V243Q-R	TTTCCGTTCACAATTAACG
10	Cystein (C)	V243C-F	AGCATCACCGTGCATCGAAGCCATTG
		V243C-R	TTTCCGTTCACAATTAACG
11	Glycine (G)	V243G-F	AGCATCACCGGGCATCGAAGCCA
		V243G-R	TTTCCGTTCACAATTAACGTATC
12	Proline (P)	V243P-F	AGCATCACCGCCGATCGAAGCCATTG
		V243P-R	TTTCCGTTCACAATTAACG
13	Alanine (A)	V243A-F	AGCATCACCGGCGATCGAAGCCA
		V243A-R	TTTCCGTTCACAATTAACGTATC
14	Isoleucine (I)	V243I-F	AGCATCACCGATTATCGAAGCCA
		V243I-R	TTTCCGTTCACAATTAACGTATCATAC
15	Leucine (L)	V243L-F	AGCATCACCGCTGATCGAAGCCATTG
		V243L-R	TTTCCGTTCACAATTAACGTATC
16	Methionine (M)	V243M-F	AGCATCACCGATGATCGAAGCCA
		V243M-R	TTTCCGTTCACAATTAACGTATC
17	Phenylalanine (F)	V243F-F	AGCATCACCGTTTATCGAAGCCA
		V243F-R	TTTCCGTTCACAATTAACGTATC
18	Tyrosine (Y)	V243Y-F	AGCATCACCGTATATCGAAGCCATTG
		V243Y-R	TTTCCGTTCACAATTAACG

19	Tryptophan (W)	V243W-F	AGCATCACCGTGGATCGAAGCCATTG
		V243W-R	TTTCCGTTCACAATTAACG

[a]Desired amino acid at position V243 of wild-type CtLac.

Table S2 Real-time measurement of dissolved oxygen consumption rate (μM O_2/min) by wild-type CtLac (WT), V243D, and V243M in the presence of different concentrations of 2,6-DMP ranging from 100 - 1000 μM at 25℃ and pH 8.0.

Concentration of 2,6-DMP (μM)	WT	V243D	V243M
100	0.81	2.1	1.0
200	1.5	2.0	0.8
300	2.5	3.4	0.6
400	3.2	4.0	0.7
500	2.4	4.2	0.6
600	2.9	4.9	1.5
700	3.9	4.4	1.4
800	4.3	4.5	1.3
900	3.9	5.2	1.5
1000	4.2	5.8	0.9
